# Promoting Healthy Organizations Through Urban Nature: Psychological and Physiological Effects in Healthcare Workers

**DOI:** 10.3390/ejihpe15080159

**Published:** 2025-08-14

**Authors:** Norida Vélez, Diana Marcela Paredes-Céspedes, Angélica Cruz-Pérez, Ronald López, Alejandra Parada-López, Eliana M. Téllez-Ávila, Paola Rodríguez de Silva, Ana Munevar, Diana Marcela Rodríguez González, Paola Fuquen, Juan Carlos Santacruz, Jeadran Malagón-Rojas

**Affiliations:** 1 Grupo de Salud Ambiental y Laboral, Dirección de Investigación en Salud Pública, Instituto Nacional de Salud, Bogotá C.P. 111321, Colombia; nvelez@ins.gov.co (N.V.); mariacruz1730@gmail.com (A.C.-P.); rlopez@ins.gov.co (R.L.); mparada@ins.gov.co (A.P.-L.); etellez@ins.gov.co (E.M.T.-Á.); jmalagon@ins.gov.co (J.M.-R.); 2 Jardín Botánico de Bogotá José Celestino Mutis, Bogotá C.P. 111071, Colombia; paola.rodriguez@jbb.gov.co (P.R.d.S.); ana.munevar@jbb.gov.co (A.M.); 3 Área de Seguridad y Salud en el Trabajo, Unidad de Talento Humano, Hospital Militar Central, Bogotá C.P. 110231, Colombia; drodriguez@homil.gov.co (D.M.R.G.); pfuquen@homil.gov.co (P.F.); 4 Fundación Colombiana del Corazón, Bogotá C.P. 110121, Colombia; jcsantacruz2012@gmail.com

**Keywords:** forest therapy, cortisol awakening response, stress, health workers, nature immersion, sleep quality, anxiety, fatigue

## Abstract

Background: Healthcare professionals experience high levels of stress due to demanding work, especially in metropolitan areas. Nature-based interventions offer potential mental health benefits. This randomized intervention study aimed to evaluate the effects of nature immersion therapies on mental health outcomes in healthcare workers with different psychological risk in Bogota, Colombia. Methods: During a period of 6 months, a total of 82 healthcare workers from two institutions were assigned to three groups: two exposed weekly to nature (parks and forests) and one control group with monthly conventional interventions. Psychological assessments of stress, anxiety, fatigue, and sleep quality were conducted at three time points (baseline, three, and six months of intervention). Cortisol Awakening Response (CAR) was measured monthly using immunoassay. Results: A decrease in the proportion of participants reporting high levels of perceived stress was observed in both intervention groups. Both forest and parks interventions significantly reduced anxiety and fatigue, while sleep quality improved only in the forest group. Multivariate analysis found a negative association between fatigue and forest intervention, as well as significant differences in CAR concentrations across groups over time. Conclusions: This study provides evidence that nature immersion therapy, particularly urban forests, positively impact mental and physical health, reducing stress, anxiety, fatigue, and CAR levels, and could be considered as an effective intervention to enhance workers’ resilience to stress, benefiting their overall health and well-being.

## 1. Introduction

The term *Shinrin-Yoku*, translating to “forest bathing,” has garnered significant interest following research conducted in China and Japan (Clarke et al., 2021). These studies underscore various physiological and psychological health benefits associated with this practice, which involves establishing a sensory connection with nature (Lim et al., 2020; Smyth et al., 2020). In Japan, this experience has evolved into a public health policy aimed at mitigating accumulated stress levels among workers in a predominantly work-oriented environment with limited options for work–life balance (Li, 2022).

*Shinrin-Yoku* is contextualized as a form of Nature Therapy, defined as a set of practices intended to achieve ‘preventive medical effects’ through exposure to natural stimuli that induce a state of physiological relaxation and promote health (Li, 2022). This approach identifies individuals with stress levels and seeks the “restorative effects” of nature in places such as parks, forests, or even flowers contact—with the hypothesis of enhancing physiological relaxation, strengthening immune function, and facilitating recovery (Paredes-Céspedes et al., 2024). These natural responses are integrated into the Evidence-Based Medicine model, contributing to the achievement of preventive medical effects.

From a psychological perspective, effects encompass the influence of psychological trauma on mental health, affecting both individuals and communities (Silove et al., 2008). These may manifest as anxiety, depression, mood swings, cognitive distortions, stress, or even post-traumatic stress disorder (PTSD), significantly impacting overall well-being and daily functioning (Pratiwi et al., 2024; Shirazi et al., 2025; Stier-Jarmer et al., 2021; Takayama et al., 2022). In this context, nature immersion therapies have been shown to positively regulate well-being by reducing stress, depression, and anxiety. Additionally, these therapies have been associated with improvements in both sleep quality and duration (Andersen et al., 2021; Pratiwi et al., 2024; Shirazi et al., 2025; Stier-Jarmer et al., 2021; Takayama et al., 2022).

On the other hand, physiological effects refer to any change or alteration that occurs in an individual’s body as a result of physical, mental, or medical intervention. These effects can be measured and observed through various biological markers and include changes in bodily functions, hormone levels, or other physiological responses (Noushad et al., 2021). In this regard, studies have demonstrated the benefits of nature immersion therapies on the immune system (increased natural killer cells (NK) and cancer prevention), and in the cardiovascular system (reduced hypertension) (Andersen et al., 2021; Chae et al., 2021; Stier-Jarmer et al., 2021). These interventions have also been associated with the regulation of cortisol levels, a key biomarker of the hypothalamic–pituitary–adrenal (HPA) axis and stress response, where lower cortisol concentrations have been related to reduced psychological distress and improved mental health (Dziurkowska & Wesolowski, 2021). A more specific marker of cortisol dynamics is the Cortisol Awakening Response (CAR), the rapid surge in cortisol concentrations within the first 30–45 min after awakening. CAR plays a crucial role in preparing the human organism to face daily challenges and is a sensitive indicator of psychological well-being (Law & Clow, 2020). The dysregulation of CAR can reflect disruptions in stress management and is associated with mental health challenges (Schulz et al., 2023). These disruptions highlight the complex interaction between physiological processes and mental health.

The existing literature has explored the effects of nature immersion therapies in a variety of professional settings (Kavanaugh et al., 2022; Razai et al., 2023). Our research group conducted a systematic review to evaluate the available evidence on the effects of forest therapies on stress, anxiety, and depression levels (Paredes-Céspedes et al., 2024). Throughout the review, we found and assessed these nature-based therapies in different populations, including adolescents, apparently healthy individuals, elderly people, and patients diagnosed with various diseases, primarily cardiovascular or neurological conditions, as well as oncology patients (Keller et al., 2023; Li, 2022; Peterfalvi et al., 2021).

Our research group conducted a systematic review to evaluate the available evidence on the effects of forest therapies on stress, anxiety, and depression levels. Although this meta-analysis included only studies involving populations without diagnosed chronic diseases or risk factors such as hypertension, the results were inconclusive and did not provide definitive evidence on the impact of these therapies on mental well-being (Paredes-Céspedes et al., 2024).

The lack of conclusive evidence is mainly due to the limited number of studies with rigorous methodologies, small sample sizes, short intervention durations, and unclear group assignment processes. These limitations highlight the need for more robust research to produce reliable data (Paredes-Céspedes et al., 2024).

In the occupational context, research suggests that *Shinrin-Yoku* and other nature-based interventions can improve job performance across various professional settings (Choi et al., 2022; Y. Kim et al., 2022; Rajcani et al., 2021).

However, burnout in healthcare professionals shows unique challenges due to the emotional toll of caring for patients in pain, which may lead to compassion fatigue and negatively impact their physical, emotional, and spiritual well-being (Razai et al., 2023). Studies indicate a high prevalence of mental health problems among healthcare workers, evidenced by significantly higher rates of work absenteeism due to burnout or stress-related disabilities (Salari et al., 2020). Additionally, demands in hospital settings have intensified in recent years, particularly during the COVID-19 pandemic, leading to an increased workload for healthcare staff (Santacruz & Blandón, 2020; Zhu et al., 2020). Therefore, it is essential to identify these stressors and develop effective interventions to enhance workers’ resilience to stress, thereby improving their productivity and overall health and well-being.

A 2022 study, assessing the impact of a *Shinrin-Yoku* (forest bathing) intervention on physician/healthcare professional burnout took place. Participants were randomized into an intervention group and a control group. Although no significant differences were found between pre- and post-test scores, subjective responses indicated reduced stress and improved well-being following the intervention. This study suggests that while objective physiological measures may not yet fully capture the benefits of *Shinrin-Yoku*, participants’ perceived improvements highlight its potential as a stress-reduction strategy (Kavanaugh et al., 2022).

Globally, it is estimated that three out of five people (an average of 62% across 31 countries) have experienced stress severe enough to impact their daily lives at least once (Ipsos, 2024). Work is identified as the leading cause of stress worldwide (24%), followed by financial insecurity (21%) (WIN, 2024). In Colombia, a survey by the Ministry of Health and Social Protection found that 66.3% of the population has faced a mental health issue at some point, with a significantly higher prevalence among women (69.9%) (Ministerio de Salud y Protección Social, 2023).

Similarly, the Ministry of Labor reported that 80% of workers in the country experience high levels of job-related stress, reflecting a disconnect between working hours and actual efficiency, with work overload being a major contributing factor. According to the Organisation for Economic Co-operation and Development (OECD), Colombia ranks among the countries with the longest working hours worldwide; however, this does not necessarily translate into higher productivity (Bernal, 2024).

In Latin-American countries like Chile and Colombia, the application of nature immersion therapies and their effects on health have become popular in recent years, to the extent that adapted guidelines have been developed for individuals interested in engaging in these health practices in nature (Santacruz & Blandón, 2020; UTEM, 2020). However, to date, there are no studies supporting the use of these therapies and evaluating their impact on physiological and psychological biomarkers in our country. Therefore, this study aims to assess the effect of nature immersion therapies on stress levels in healthcare workers in Colombia.

## 2. Materials and Methods

The study received approval from the Ethics and Research Methodologies Committee (CEMIN) of the National Institute of Health in Colombia (021-2022), the Ethics Committee of the District Secretariat of Health (CIESDSCTI20210017), and the Research Ethics Committee of the Central Military Hospital (2022170). Additionally, the study was registered in the ClinicalTrials.gov registry (NCT05315388) in the US National Library of Medicine.

### 2.1. Study Design and Population

An analytic and intervention study (Ranganathan & Aggarwal, 2019) was carried out in healthcare workers in Bogota, Colombia. Participants were recruited from the Central Military Hospital (institution A) and the National Institute of Health (institution B). The target population consisted of healthcare workers who lived in Bogota and were preselected based on their psychosocial risk in the workplace. To identify the study population, meetings were held with the human resources department and the occupational health and safety group of each institution, who provided information on healthcare workers at psychosocial risk. Subsequently, visits were made to each department, during which the project’s objectives were explained, and individuals were invited to participate.

#### Eligibility Criteria

Health care workers, between 18 and 60 years, working in operative and administrative departments, were included considering their psychosocial risk assessment results according to the Epidemiological Surveillance Program (a structured system designed to monitor and analyze occupational health-related risks, facilitating early identification and intervention strategies to protect workers’ well-being) in each institution. Students, radiology service workers, pregnant women, and individuals with untreated chronic illnesses where excluded. After receiving a voluntary invitation to participate in the project, those who agreed signed an informed consent form outlining the benefits and their right to leave the study at any time. Once the worker agreed to participate in the study, a code was assigned to conduct randomization, ensuring homogeneity in-group stratification.

Three groups were established in accordance with an intervention group in metropolitan parks, a second intervention group in urban forest, and finally, the control group. A validated sociodemographic survey was then applied to all participants, encompassing demographic characteristics, occupational variables, lifestyle, dietary habits, and perceived health information. Participants underwent a medical examination to assess their health status.

The assignment of participants to the groups was performed randomly, based on baseline salivary cortisol concentrations. To ensure an equitable distribution, a random number table was used, minimizing potential bias in group allocation (Berger et al., 2021). The randomization process was carried out by a designated team member who had access only to the cortisol data and participant codes, while the researchers remained blinded to this process to maintain objectivity.

### 2.2. Intervention Development

Each participant received detailed training on how to complete the instruments, as well as the procedures for collecting saliva and blood samples. Additionally, a group was formed on a messaging platform to facilitate communication, where reminder messages were sent regarding sample collection dates, designated therapy locations, and pickup points for transportation to urban parks and forests.

Participants in the control group received tailored intervention activities for six months, based on the results obtained from the psychosocial risk diagnostic report and in alignment with the institutional intervention plan for each institution in 2023. This group participated in training sessions covering topics such as leadership, interpersonal relationships, teamwork, emotional intelligence, and adaptation to change. Additionally, this group received a weekly set of digital content designed exclusively for them, encompassing themes related to the interventions, including managing interpersonal relationships, Neuro-Linguistic Programming, habit formation, and mindfulness.

The Intervention Groups underwent a minimum of 17 nature therapy sessions between May and December 2023, meeting once a week for six months, with each session lasting one hour. These sessions were facilitated by trained Nature Guides, part of the Nature, Health and Culture Program at the Botanical Garden of Bogota.

The natural areas selected for the intervention were chosen based on criteria such as biodiversity, tree density, tree cover, accessibility, wildlife presence, minimal human intervention, low noise pollution, and access to public services. A total of three natural areas were selected for metropolitan parks (Simon Bolivar, Los Novios, and the Botanical Garden of Bogota), and three for urban forests (Entrenubes, Las Mercedes, and La Florida). Participants rotated weekly among the selected natural areas according to their assigned group.

The intervention phases followed the typical forest therapy model, including environmental observation and recognition moments, passive walks, mindful breathing, sensory awareness, and experiential components. All nature immersion sessions took place in the morning. During each intervention, the temperature (°C) and relative humidity (%) of the natural environment were measured every 15 min from the beginning to the end of each session using a thermo-hygrometer with a data logger (Shenzhen Yowexa Measurement Technology Co., Ltd., Guangdong, China).

### 2.3. Measurement Tools for Psychological Evaluation

Perceived Stress Scale-14 (PSS-14): this scale is widely used for assessing stress levels (Campo-Arias et al., 2009). This instrument has been validated in Spanish-speaking countries, including Colombia. Each question presents four response options (“0 = never, 1 = almost never, 2 = sometimes, 3 = fairly often, 4 = very often”), resulting in a cumulative score ranging from 0 to 40, where a higher score indicates a greater level of stress (Campo-Arias et al., 2009).

Yoshitake Subjective Fatigue Scale: the Fatigue Symptoms Questionnaire consists of 30 items subdivided into three scales (Vega-Valero et al., 2019). The first scale measures general fatigue symptoms (items 1–10), the second measures physical fatigue (items 11–20), and the last measures mental fatigue (items 21–30). Participants respond to five options (never, almost never, seldom, often, almost always, and always). Scoring ranges from 0 to 5, with 0 indicating “never” and 5 indicating “always” (Vega-Valero et al., 2019).

Anxiety Level Perception Measurement: the State-Trait Anxiety Inventory (STAI) consists of two self-assessment scales that measure two independent concepts of anxiety: state and trait (Conde et al., 2009). State anxiety is defined as a transient emotional condition characterized by subjective feelings of tension and apprehension. Trait anxiety, on the other hand, refers to a stable and enduring aspect of an individual’s personality, reflecting the general tendency to respond to a variety of situations with anxiety. Both the state and trait scales contain 20 items each, scored on a Likert-type scale with four response options (from 0 to 3) (Conde et al., 2009). Higher scores on the trait anxiety scale indicate a greater predisposition toward experiencing anxiety across different contexts.

Pittsburgh Sleep Quality Index (PSQI): a widely utilized and validated method, it comprises 19 self-evaluation items that analyze different determinants of sleep quality, grouped into seven components: sleep quality, sleep latency, sleep duration, sleep efficiency, sleep disturbances, use of sleep medication, and daytime dysfunction (Escobar-Córdoba & Eslava-Schmalbach, 2005). Each component is scored from 0 to 3. The total score of the PSQI, which ranges from 0 to 21 points, is derived from the sum of the seven components, with a higher score indicating a poorer sleep quality (Escobar-Córdoba & Eslava-Schmalbach, 2005).

Measurements from the psychological scales were conducted three times during the research: at the study initiation, three months into the intervention, and at the sixth month of the research. All scale applications were self-administered, carried out in the presence of the research team to address any questions that may arise.

### 2.4. Salivary Cortisol Determination for Physiological Evaluation

Saliva samples were self-collected by participants using Salivette^®^ tubes (Sarstedt International, Nümbrecht, Germany), following the provided instructions. To ensure accurate sample collection, the research team gave detailed instructions to each participant, emphasizing the potential impact on results if information was not recorded correctly. Participants were also instructed to label the collection time as soon as the self-sampling process began. Participants answered questions about the exact awakening and sleep time. This information was considered if atypical values were presented. Samples were centrifuged at 1459× *g* for 10 min and stored at −70 °C until to analysis. Seven salivary cortisol measurements were collected prior to the interventions and monthly over a six-month period. After obtaining two samples spaced 45 min apart, the labeled tubes were submitted to the research team.

Cortisol levels were quantified using the QuicKey Pro Human Cortisol ELISA Kit (Elabscience, Houston, TX, USA), following the standard protocol, and reading the plate at 450 nm. The CAR value was calculated as the difference between the wake-up sample (1st sample) and the sample collected 45 min later (2nd sample). We considered various measurements and strategies in accordance with the consensus guidelines published by Stalder et al. (2016, 2022) for assessing the CAR (Table S1). The items considered included a self-report diary system, sensitivity analyzes using a strict accuracy margin, clear instructions about unwanted morning behavior, and state and trait-like covariates.

### 2.5. Statistical Analysis

Descriptive statistics were tabulated as arithmetic mean (standard deviation, SD) or median (interquartile range, IQR) or percentages (proportion), as appropriate. A comparison of means was performed using ANOVA or the Kruskal–Wallis test, depending on the normality of the variable, to compare the three study groups. The Wilcoxon test was used to evaluate paired differences between baseline and final measurements for each variable. Categorical variables were analyzed using the chi-square test. Regarding the psychological variables, a multivariate analysis of variance (MANOVA) (De Melo et al., 2022) was performed, the levels of significance were evaluated based on Wilks’ lambda, followed by a multivariate regression analysis of the psychological variables, including the interaction between the intervention group and the institution, with adjustment for sex. Since CAR concentrations were measured repeatedly throughout the intervention for all three study groups, a multilevel mixed-effects linear regression model was employed to analyze the data (Tomiko Yamada Da Silveira et al., 2023). All CAR measurements were considered as a depend variable, with time treated as a continuous variable. The selection of variables to include in the multivariate model was performed using the Lemeshow–Hosmer criterion, considering those that presented a *p*-value < 0.20 in the bivariate analysis. This approach allows for the identification of potentially relevant predictors for the final model, ensuring adequate explanatory power and avoiding the premature exclusion of variables that could be significant in a multivariate context (Table S1) (Hosmer & Lemeshow, 2000).

As part of the statistical analysis, an evaluation of predictive margins was conducted (Hu et al., 2023). This approach enabled specific comparisons of estimated trends in CAR concentrations across the study groups over time, accounting for the repeated measures structure of the data. Additionally, the slopes for each study group were derived from the analysis, providing insights into the specific patterns of change in CAR concentrations during the intervention (Law & Clow, 2020). A *p*-value < 0.05 was considered statistically significant. All analyzes were performed using GraphPad Prism 9 (RRID: SCR_002798), and Stata 15.0 (RRID:SCR_012763).

## 3. Results

**Figure 1:**
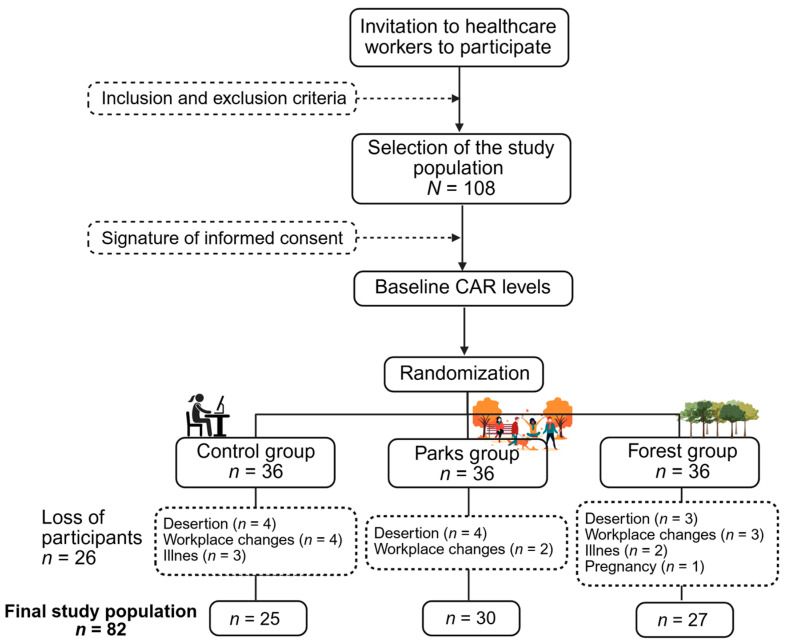
A flow diagram of the study population selection.

A total of 108 participants were included in the study. During the research, 26 participants were discarded due to reasons such as desertion, workplace changes, illness, and other personal factors. Consequently, the final number of participants was 82, divided into three groups: 34.09% (*n* = 30) for the metropolitan parks group, 30.68% (*n* = 27) for the urban forest group, and 28.40% (*n* = 25) for the control group (Figure 1), with a response rate of 75.93%. In Table 1, the main sociodemographic variables of the participants are shown. Overall, no significant differences were found between groups for any of the evaluated sociodemographic characteristics (*p* > 0.005). However, a high predominance of female participants was observed across all three study groups, exceeding 70%. This finding aligns with national statistics, which report that 80% of healthcare professionals are women (Ministerio de Salud y Protección Social, 2023). Additionally, the age of participants was between 22 and 66 years, with a mean in the body mass index (BMI) of 24.36 ± 3.99 kg/m^2^. Regarding harmful habits, only 3.66% of the population were active smokers, and 25.35% reported alcohol consumption. Nevertheless, more than 50% of the study population engaged in physical activity.

The study included healthcare professionals based on the World Health Organization’s definition (WHO, 2022), covering a range of occupations as follows: psychologists (*n* = 5), nurses (*n* = 22), laboratory technicians (*n* = 15), biologists/bacteriologists (*n* = 27), therapists (*n* = 4), chemists (*n* = 3), veterinarians (*n* = 3), and physicians/dentists (*n* = 3).

### 3.1. Psychological Effects

**Figure 2:**
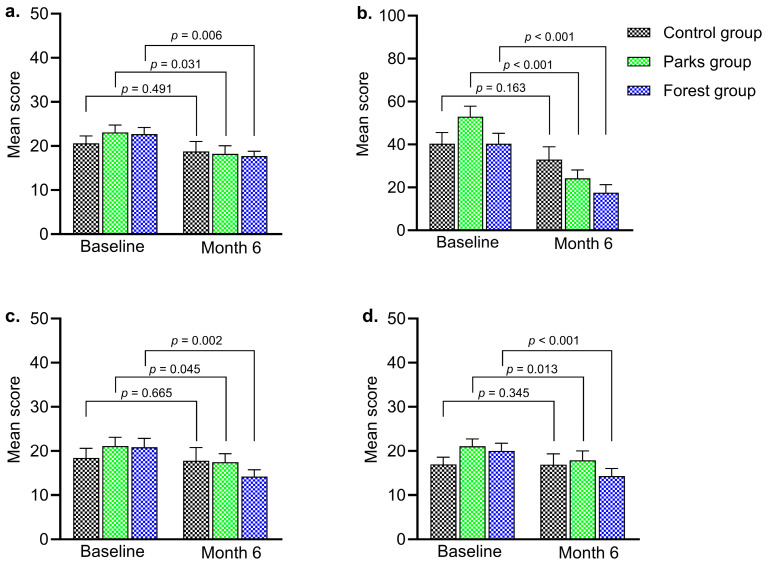
A comparison of work-related fatigue levels in the study population between the initial and final measurements. (**a**). Perceived stress scale (PSS-14), (**b**). Yoshitake’s subjective fatigue, (**c**). The state anxiety, and (**d**). The trait anxiety. *p* values were obtained using the Wilcoxon signed-rank test. *p* < 0.05 was considered statistically significant.

Regarding the PSS14 scale, all three groups showed a reduction in the percentage of participants perceiving high stress between the first and third assessments. In the parks group, high-stress levels decreased from 30% to 16.6%, whereas in the forest group, they declined from 25.9% to 7.4%. The control group showed a smaller decline, starting at 16% and ending at 12%. Notably, participants in the parks group experienced a significant increase in low-stress levels, rising from 16.6% to 46.6% (changing from high to low stress level). These findings suggest positive changes in stress perception over the course of the intervention. Statistical analysis demonstrated significant differences in scores between the parks (*p* = 0.0312) and forests (*p* = 0.0063) (Table 2 and Figure 2a).

Data from the Yoshitake scale indicated positive changes in the intervention groups, with a significant decrease in the number of participants reporting general fatigue as the interventions progressed (*p* < 0.0001). Both groups showed significant improvements, with reductions in the number of participants experiencing physical and mental fatigue (Table 2 and Figure 2b). In contrast, no positive changes were found in the control group.

The state anxiety results indicate positive effects from the interventions in the park and forest groups. In general, the parks group showed a decrease in high-stress levels from 23.3% to 16.6%, while the forest group decreased from 22.2% to 3.7%. Regarding trait anxiety, the control group remained stable throughout the intervention (*p* = 0.345), while both the parks and forest groups demonstrated advantages, with a higher number of participants experiencing low levels of trait and state anxiety. At 6 months, significant differences were found between the intervention groups: for the parks group, with *p* = 0.045 and *p* = 0.013, and for the forest group, with *p* = 0.002 and *p* = 0.0005, for state and trait anxiety, respectively (Table 2 and Figure 2c,d).

Regarding sleep quality, distinct patterns emerged across the groups. Both the parks and control groups showed no changes, with 66% and 56% of participants, respectively, reporting poor sleep quality throughout the study. In contrast, the forest group showed improvement, with the percentage of participants reporting poor sleep quality decreasing from 71% from the beginning of the intervention to 56% to the end. Although positive trends in sleep quality were observed in the forests group, statistical analysis revealed no significant differences (Table 2). None of the psychological parameters evaluated differed significantly based on the physical activity reported by the study population.

Additionally, we evaluated whether environmental conditions (temperature and humidity) influenced participants’ perceived well-being and, consequently, their psychological outcomes. Although significant differences in temperature and relative humidity were observed in both intervention groups over the 24-week intervention period (Figure S1), no association was found between these environmental conditions and stress, anxiety, fatigue, or sleep quality in either intervention group.

A significant difference was identified in the MANOVA analysis, which included the results from the four psychological instruments assessed at the end of the intervention (*p* = 0.05). The model was adjusted for sex and the interaction between the influence of the intervention group and the institution where participants were employed (*p* = 0.001). Based on these results, a multivariate regression analysis was performed, indicating a positive association for females compared to males in sleep quality (coefficient = 2.26; *p* = 0.036) and state anxiety levels (coefficient = 6.74; *p* = 0.045). Regarding the interaction between the intervention group and the institution, a negative association was identified for general fatigue in the interaction between the urban forest intervention group and actively working at institution B (coefficient = −25.70; *p* = 0.024). (Table 3).

### 3.2. Physiological Effects

Additionally, although the mean difference analysis did not show a significant change in CA, the CAR levels in saliva were measured monthly throughout the intervention, including the baseline and the six-month follow-up. We observed that all three groups started with similar cortisol levels; however, variations were observed from the third CAR measurement onward. An increase in CAR levels was observed in both intervention groups (parks and forests) compared to the control group, which maintained stable values throughout the intervention period. Notably, CAR levels showed two significant peaks during the third and fifth months of the intervention, with a more pronounced increase in the metropolitan parks group. This pattern suggests that the urban forest environment may have been more effective in reducing stress. These increases can be partially attributed to significant work-related changes that took place during these months at the two institutions to which the study population belonged. These changes included the termination of employment contracts for a large portion of the workforce, leading to an increased workload for the remaining employees. Additionally, there were changes in the executive personnel, generating uncertainty and concern among workers. It is important to note that the intervention groups were more affected by these changes, as the control group was predominantly composed of professionals serving as department heads, holding permanent contracts, and whose daily routines and work schedules were not significantly affected.

**Figure 3:**
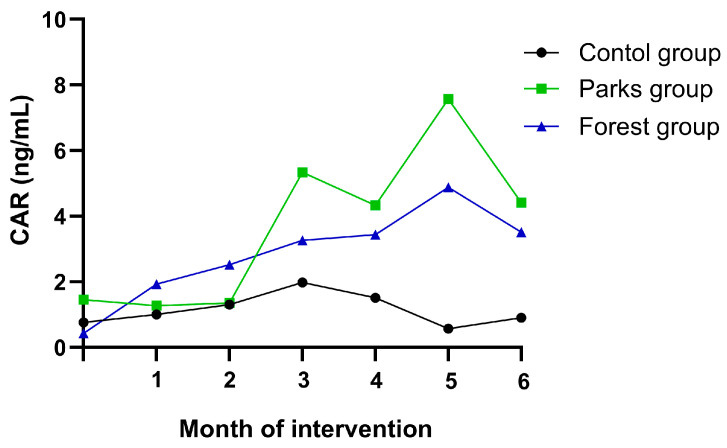
Cortisol awakening response (CAR) levels in saliva samples during a six-month intervention between study groups.

In the control group, cortisol levels significantly increased in the third month and then decreased in subsequent weeks, without showing a sustained reduction, reflecting the lack of intervention in this group (Figure 3).

In relation to the multivariate model, a mixed-effects multilevel linear regression was performed, which demonstrated a significant difference in CAR concentrations across the three study groups over time [control group: coefficient = 1.20, *p* = 0.03; parks group: coefficient = 1.70, *p* < 0.01, and forest group: coefficient = 1.74, *p* = 0.01]; after adjusting the model for the institution to which each individual belonged, educational level, and sex (Table 4).

**Figure 4:**
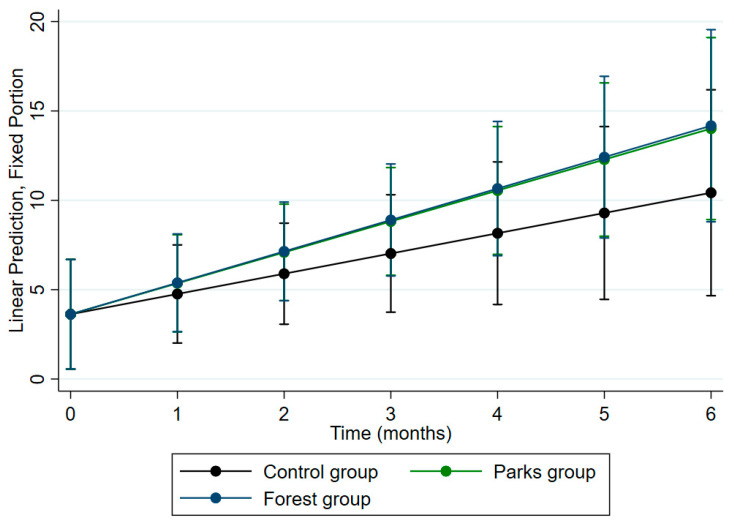
An adjusted prediction of CAR concentrations throughout the intervention for the study groups according to the multilevel mixed-effects linear regression model.

Additionally, the analysis of predictive margins revealed that the CAR behavior in the intervention groups showed a different slope compared to the control group (Figure 4), suggesting that nature immersion therapies reflect a difference in cortisol modulation upon awakening compared to the therapies regularly conducted in the institutions for psychosocial risk management.

## 4. Discussion

Our research suggests that exposure to natural settings, urban forests, and metropolitan parks, can contribute to improved mental and physical well-being by alleviating stress, anxiety, and overall fatigue. To our knowledge, this study is the first of its kind to be conducted for periods longer than two months under real-life conditions, providing valuable information on the medium-term effects of psychological and physiological parameters such as stress, fatigue, and anxiety levels, as well as sleep quality and CAR levels.

Previous studies have shown that continuous stress, anxiety, workplace fatigue, and poor sleep quality faced by healthcare professionals can negatively affect their psychological well-being. This can hinder their professional performance and have a significant impact on the quality of care they provide to patients (Huang et al., 2022; Kweon et al., 2024; Rink et al., 2023). Strategies aimed at providing emotional and psychological support to healthcare staff often emphasize individual behaviors, such as practicing yoga, maintaining proper nutrition, and ensuring sufficient sleep. However, the healthcare system’s attention to organizational health is inherently connected to the overall functioning of the system (both organizational and individual well-being), which must be addressed through a coordinated approach that acknowledges this interconnection.

An ideal approach to well-being should encompass benefits for individuals as distinct entities while simultaneously fostering a sense of community and belonging within a broader system. In response to this need, numerous methods and instruments have been developed to identify and assess psychosocial conditions and their impact on workers’ health and well-being. Over four decades of research, evidence has increasingly documented the extent of mental health benefits associated with a connection to nature (Hansen et al., 2017). This has fueled interest in practices such as “*Shinrin-yoku*”, or forest bathing, also known as nature immersion therapies (Rajoo et al., 2020). In the current study, we observed positive changes following a six-month intervention in groups exposed to natural environments, with results reflected in the psychological, social, and physiological well-being of healthcare workers.

Several studies have shown that spending time in nature improves mental health (Kotera et al., 2022; Lim et al., 2020; Smyth et al., 2020). In our research, groups exposed to nature (metropolitan parks and urban forests) demonstrated a significant reduction in stress levels, with the forest group showing a greater decrease in perceived stress, likely attributable to the immersive and secluded nature of this environment. Similarly, a reduction in state anxiety was observed among these groups, whereas the control either remained unchanged or showed an increase in trait anxiety. These findings suggest that interaction with natural environments may positively contribute to stress perception, aligning with previous studies linking nature contact to benefits in emotional regulation and reduced hypothalamic–pituitary–adrenal (HPA) axis activity (Lopresti et al., 2022). Additionally, natural environments have been demonstrated to improve cognitive performance and restore an individual’s ability to focus and pay attention clearly (Rhee et al., 2023). Therefore, natural settings may provide an environment conducive to disconnecting from daily stressors, thereby facilitating more effective emotional regulation.

Some studies have described that contact with nature promotes more effective physical, mental, emotional, and restorative recovery, as well as its positive impact on job performance by strengthening individual capacities (Yang et al., 2023; Zabini et al., 2020). This aligns with the findings in our study, where participants who interacted with natural environments reported improvements in fatigue, reinforcing the concept that nature acts as a restorative resource, reducing physical and emotional overload. We observed positive changes in sleep quality within the forest group, suggesting that the isolation and reduction of urban stimuli provided by forest settings may have influenced these changes. However, the lack of statistical significance highlights the need for further studies to explore other confounding factors, such as prior sleep patterns or nocturnal habits.

A 2019 United Kingdom (UK) study included nearly 20,000 people where they examined associations between contact with nature, self-reported health, and well-being (White et al., 2019). The authors described that spending at least 120 min a week in nature improved participants’ perceived health and well-being, and furthermore that time was unrelated to a prolonged immersion experience or several brief experiences in nature. This research concludes that contact with nature intertwines individual well-being with empowerment and autonomy, expanding awareness of participating in a cooperative and reciprocal ecosystem.

In a study conducted on actively employed individuals who experienced an increase in workload intensity or changes in their work dynamics due to the COVID-19 pandemic, a nature-based intervention was implemented. This intervention included trail walks, foot immersion in thermal waters, meditation accompanied by forest sounds and music, the use of aromatic oils on the hands, stretching exercises, and the sharing of personal experiences (Kweon et al., 2024). The results showed a significant reduction in perceived stress, depression, and anxiety levels, as well as improvements in sleep quality and participants’ resilience, observed at the conclusion of the intervention. These findings underscore the effectiveness of nature-based therapies in improving sleep quality and alleviating work-related stress. Kweon et al. highlighted that the benefits of forest therapy were consistently observed across all professional groups included in the study, demonstrating its broad applicability regardless of specific occupational challenges.

On the other hand, stress levels can be evaluated through the physiological levels of different biomarkers, including adrenaline, primarily associated with mental stress; noradrenaline, linked to physical stress; and cortisol, which can indicate both types of stress (James et al., 2023; Knezevic et al., 2023). In this study, we determined the CAR concentration based on the salivary levels. The results showed fluctuations, particularly in the third measurement, which could be influenced by uncontrolled factors, such as lifestyle, increased workload related to the city’s second respiratory peak (occurring between September and October), and even organizational changes related to the termination of employment contracts experienced by participants during the intervention. Despite this, the decrease in cortisol levels that was observed in the forest group suggests a more consistent positive impact of the natural environment on the physiological response to stress.

Moreover, the lack of an objective measurement of wake-up time (e.g., actigraphy, and polysomnography) may lead to inaccurate data estimates, as this study relies solely on participants’ self-reports and photographs of the sample collection time. Although these objective measurements are recommended in the guidelines by Stalder et al. (2016, 2022), their implementation in longitudinal clinical and intervention studies is challenging due to limited equipment availability and high costs. Furthermore, prolonged use of these devices may cause discomfort among participants, potentially affecting adherence and data accuracy. These challenges represent a significant limitation in most studies that use the CAR as an outcome (Giglberger et al., 2022; Nasser et al., 2023; Torres et al., 2024).

A spearman correlation analysis between the PSS-14 assessment and CAR concentrations revealed no significant correlation between these two variables that would allow for an estimation of stress in the study population across different dimensions. This may suggest that the participants’ perception of their stress level does not align with their physiological state. Studies have documented the absence of a relationship between these two approaches to stress assessment, partly because hormonal responses vary according to individual and contextual factors, leading to discrepancies between subjective measures and biological stress markers (Bani-Issa et al., 2020; Canuto et al., 2021). Additionally, the PSS-14 assesses the extent to which life situations are perceived as unpredictable, uncontrollable, and overwhelming over a one-month period, which may be too short to induce lasting changes in the regulation of cortisol secretion by the HPA axis (Mikkelsen et al., 2017). This may explain the non-significant correlation observed in this study. Another possible explanation is that the PSS-14 evaluates perceived stress over the past month, while salivary cortisol levels are measured at a single time point each month. As a result, these assessments capture stress within different time frames (Canuto et al., 2021).

In contrast, all psychological instruments showed a high correlation with one another in the measurements taken at the end of the intervention. The findings from the multivariate analysis indicated that, for the psychological variables, women exhibited a significant positive association with state anxiety levels, general fatigue, and sleep quality, compared to men. Furthermore, the intervention group in urban forests and working at institution B showed a significant reduction in general fatigue levels compared to the control group. Regarding multivariate regression analysis, underscoring the impact of nature immersion therapies on cortisol modulation, the mixed-effects multilevel linear regression revealed a significant difference in CAR concentrations across the three study groups over time, even after adjusting for the institution to which participants belonged. Furthermore, the distinct slope patterns in CAR behavior observed in the intervention groups emphasize the efficacy of nature immersion therapies in influencing cortisol dynamics. These findings suggest the potential of integrating nature-based approaches into health and wellness programs to address psychosocial stressors more effectively.

A study conducted by B. J. Park et al. (2010) in 24 forests in Japan evaluated physiological changes in 280 participants randomly divided into groups that alternated between exposures to forested and urban areas. Each session consisted of walking and observing the landscape; saliva cortisol measurements indicated significantly lower levels in forest environments. In this sense, various studies have related nature-based therapies with a reduction in cortisol levels across different population groups, such as occupationally active personnel, children, apparently healthy individuals, and patients diagnosed with mental, cardiovascular, and other pathologies (Jezova et al., 2024; Kweon et al., 2024; Ye et al., 2023; Mygind et al., 2018); however, it is important to note that such measurements were performed only once per individual, limiting the assessment of temporal trends. In contrast, our study implemented a longitudinal design with seven measurements during the intervention, providing a broader perspective on CAR dynamics over time and under repeated conditions of exposure to nature. This further reflects the relevance of incorporating multiple measurements and prolonged monitoring to capture interactions between natural environments and stress physiology.

Additionally, most longitudinal studies based on nature therapies have been conducted over short time periods, where participants were exposed to nature experiences lasting less than three months (Keller et al., 2023; J. Kim et al., 2021; McEwan et al., 2021).

Extending the duration of these interventions is crucial to assessing whether the psychological benefits are sustained, diminished, or enhanced with prolonged and recurrent exposure. Understanding the long-term effects of nature immersion could provide valuable evidence for its integration into public health strategies, reinforcing existing workplace psychosocial risk prevention programs and optimizing their effectiveness at both regulatory and organizational levels.

It is of particular significance that, to our knowledge, this is the first study to longitudinally evaluate the influence of a nature-based intervention on CAR concentration. This emphasizes the relevance of the findings, as cortisol (stress-related-hormone) is a biomarker that exhibits a distinct 24-h rhythmicity (circadian rhythm) (O’Byrne et al., 2021), and its measurement at a single point in time does not provide a reliable result for assessing the effect of an intervention. Additionally, CAR is considered a unique aspect of the HPA axis activity, as it is not significantly related to diurnal cortisol measurements, but has been found to be associated with numerous psychosocial variables and mental health-related factors, such as variations in work-related stress levels, as well as increased fatigue and burnout (Anderson & Wideman, 2023; Dziurkowska & Wesolowski, 2021; Stalder et al., 2025).

The findings of the present study highlight the importance of the design and selection of the natural area to maximize the psychological benefits. Some research has described that by inhaling forest scents, people absorb volatile essential oils from trees known as phytoncides, which have antimicrobial properties and have also been shown to boost immunity by increasing the number of natural killer cells (Lew & Fleming, 2024; S. Park et al., 2022).

Our study has notable strengths. We were able to obtain a reasonably sized sample, considering the logistical challenges of collecting monthly saliva samples over six months of intervention in a population experiencing a high workload, as is the case with healthcare workers. Additionally, we incorporated psychological assessments at three time points, thereby strengthening the results and enabling a longitudinal analysis of both psychological and physiological components within this study population. However, there are considerable limitations. Firstly, the assessment of CAR throughout the study presented additional challenges. Secondly, although salivary cortisol concentrations are a non-invasive method, several factors that could affect their accuracy and reliability must be considered. Following the guidelines from the expert consensus on CAR evaluation published by Stalder et al. (2016, 2022), we adhered to a rigorous protocol, as detailed in Table S1. Nevertheless, a major limitation of our study was the inability to include an objective method for verifying awakening, which could introduce variability in the CAR measurements. These challenges in obtaining CAR values, together with the intra-institutional factors to which the study population was exposed during the intervention, clearly contributed to the inability to obtain conclusive results on the effect of nature-based immersion therapies on cortisol regulation. Furthermore, despite efforts to standardize the intervention, variations in the participants’ experience of nature immersion sessions and external uncontrolled factors may have influenced the results. Finally, participant loss over the course of the study and the possible influence of external stressors, which were not controlled, further limited the interpretation of our findings. Despite these challenges, the study provides valuable insights into the therapeutic effects of natural environments on stress management, anxiety, fatigue, and other health outcomes.

Based on the results of this study, future research should focus on standardizing protocols to enhance comparability across studies, as well as incorporating additional physiological biomarkers (e.g., dehydroepiandrosterone, melatonin, oxytocin, adrenaline, noradrenaline, salivary alpha-amylase, and pro-inflammatory cytokines) to more comprehensively assess the effects of nature exposure. Furthermore, the use of real-time monitoring tools, such as activity monitors or actigraphy devices, would allow for more measurements that are objective and include a verification of awakening, thereby reducing variability in CAR assessment. It is also essential to investigate the optimal frequency, duration, and intensity of nature immersion sessions to maximize their benefits. Exploring these aspects will strengthen scientific evidence and facilitate the integration of nature-based interventions into public health strategies and psychosocial risk prevention programs.

## 5. Conclusions

This study provides evidence that natural environments, particularly urban forests, may have a positive impact on mental and physical health, reducing stress, anxiety, and general fatigue. These results reinforce the importance of considering nature-based interventions as effective strategies in the field of health promotion and stress prevention in vulnerable populations such as health care workers.

## Figures and Tables

**Table 1:** Sociodemographic characteristics of participants across three intervention groups.

Parameters		Metropolitan Parks Group	Urban Forest Group	Control Group	*p*
Total population		30	27	25	
Sex [*n* (%)]	Male	4 (13.33)	5 (18.52)	7 (28.00)	0.39
	Female	26 (86.67)	22 (81.48)	18 (72.00)	
Age (years) [mean (SD)]		43.77 (10.94)	44.30 (9.50)	40.04 (9.32)	0.21
BMI (kg/m^2^) [*n* (%)]					0.22
	Underweight	1 (3.33)	1 (3.70)	--	
	Normal weight	20 (66.67)	14 (51.85)	17 (73.91)	
	Overweight	6 (20.00)	12 (44.44)	5 (21.74)	
	Obese	3 (10.00)	--	1 (4.35)	
Current Smoking [*n* (%)]		2 (6.67)	1 (3.70)	--	0.34
Alcohol consumption [*n* (%)]		11 (36.67)	13 (48.15)	6 (24.00)	
Current physical activity [*n* (%)]		15 (53.57)	15 (55.56)	12 (75.00)	
Employer institution [*n* (%)]					0.68
	Institution A	14 (46.67)	10 (37.04)	12 (48.00)	
	Institution B	16 (53.33)	17 (62.96)	13 (52.00)	
Job activity profile [*n* (%)]					0.39
	Job that primarily involves sitting	12 (42.86)	14 (51.85)	11 (68.75)	
	Job that requires a lot of walking	8 (28.57)	9 (33.33)	4 (25.00)	
	Job that involves both walking and heavy lifting	6 (21.43)	2 (7.41)	--	
	Physically demanding job	2 (7.14)	2 (7.41)	1 (6.25)	
Tenure in the current job [*n* (%)]					0.31
	0–6 months	--	1 (3.70)	--	
	>6–12 months	3 (10.71)	--	1 (6.25)	
	>12–36 months	1 (3.57)	3 (11.11)	--	
	>36 months	24 (85.71)	23 (85.19)	15 (93.75)	

Body Mass Index (BMI). Values of *p* < 0.05 were considered statistically significant. *p*-values were obtained using the Chi-square test and ANOVA test.

**Table 2:** A comparison of stress (PSS-14), anxiety (STAI), sleep quality (Pittsburgh), and work-related fatigue (Yoshitake) measurements between metropolitan parks, urban forests, and control groups.

		Experimental Group Metropolitan Parks					Experimental Group Urban Forest					Control Group				
Scale		Baseline Mean (SD)	Month 3 Mean (SD)	*p* Value	Month 6 Mean (SD)	*p* Value	Baseline Mean (SD)	Month 3 Mean (SD)	*p* Value	Month 6 Mean (SD)	*p* Value	Baseline Mean (SD)	Month 3 Mean (SD)	*p* Value	Month 6 Mean (SD)	*p* Value
PSS-14		23.27 (10.03)	19.40 (10.23)	0.03	18.43 (10.81)	0.03	23.04 (8.56)	21.38 (8.26)	>0.05	17.92 (5.95)	0.01	21.43 (9.50)	20.84 (8.85)	>0.05	19.75 (9.75)	>0.05
STAI	State	21.17 (12.12)	18.00 (10.98)	0.05	17.67 (11.58)	0.05	21.3 (11.59)	20.50 (9.82)	>0.05	14.31 (8.61)	<0.01	19.61 (11.90)	20.6 (12.28)	>0.05	18.70 (13.42)	>0.05
	Trait	21.50 (10.06)	19.70 (9.66)	>0.05	18.27 (12.64)	0.01	20.5 (10.23)	19.35 (10.62)	>0.05	14.58 (9.23)	<0.01	18.17 (8.87)	20.0 (11.55)	>0.05	17.60 (10.89)	>0.05
Pittsburgh	Sleep efficiency	10.00 (10.44)	10.00 (12.17)	>0.05	10.00 (9.84)	>0.05	9.00 (9.84)	8.66 (10.97)	>0.05	8.66 (11.55)	>0.05	7.66 (8.96)	6.33 (7.09)	>0.05	6.66 (9.07)	>0.05
	Sleep duration	10.00 (9.64)	10.00 (7.00)	>0.05	10.00 (7.93)	>0.05	9.00 (6.08)	8.66 (7.23)	>0.05	8.66 (10.79)	>0.05	7.66 (6.35)	6.33 (4.93)	>0.05	10.00 (11.31)	>0.05
	Sleep disturbance	10.00 (14.00)	10.00 (13.89)	>0.05	10.00 (13.89)	>0.05	9.00 (13.00)	8.66 (15.01)	>0.05	8.66 (14.15)	>0.05	7.66 (10.69)	6.33 (9.23)	>0.05	6.66 (9.81)	>0.05
	Sleep latency	10.00 (14.80)	10.00 (14.73)	>0.05	10.00 (15.59)	>0.05	9.00 (12.17)	8.66 (12.50)	>0.05	8.66 (14.15)	>0.05	7.66 (11.55)	6.33 (8.38)	>0.05	6.66 (9.81)	>0.05
	Sleep quality	10.00 (4.00)	10.00 (5.19)	>0.05	10.00 (2.64)	>0.05	9.00 (1.00)	8.66 (3.05)	>0.05	8.66 (5.03)	>0.05	7.66 (0.57)	6.33 (3.05)	>0.05	6.66 (2.08)	>0.05
	Use of sleep medication	10.00 (7.93)	10.00 (7.93)	>0.05	10.00 (8.71)	>0.05	9.00 (7.81)	8.66 (7.37)	>0.05	8.66 (9.86)	>0.05	7.66 (12.42)	6.33 (9.23)	>0.05	6.66 (9.81)	>0.05
	Sleep dysfunction	10.00 (10.82)	10.00 (11.36)	>0.05	10.00 (10.58)	>0.05	9.00 (9.16)	8.66 (7.09)	>0.05	8.66 (10.97)	>0.05	7.66 (8.14)	6.33 (6.02)	>0.05	6.66 (4.16)	>0.05
Yoshitake	General fatigue	55.67 (26.87)	43.33 (23.97)	>0.05	34.67 (27.76)	<0.01	40.00 (27.46)	34.23 (27.01)	0.05	16.92 (19.55)	<0.01	43.48 (28.22)	40.53 (31.18)	>0.05	33.50 (25.60)	>0.05
	Physical fatigue	32.00 (26.31)	29.33 (27.53)	>0.05	24.33 (25.69)	0.05	42.59 (30.83)	31.15 (27.90)	<0.01	15.00 (13.64)	<0.01	23.04 (22.04)	27.89 (26.58)	>0.05	19.00 (24.69)	>0.05
	Mental fatigue	34.00 (21.11)	26.33 (22.36)	>0.05	26.33 (22.36)	>0.05	31.85 (22.88)	18.85 (17.51)	<0.01	9.23 (13.24)	<0.01	23.04 (20.77)	23.6 (20.60)	>0.05	15.00 (22.59)	>0.05

Data are expressed as arithmetic mean and standard deviation (SD). *p*-values were obtained using the Wilcoxon signed-rank test. *p* < 0.05 was considered statistically significant.

**Table 3:** A multivariate regression model between psychological variables and the interaction between intervention group and institution, adjusted by sex.

Variable	PSS-14			STAI–State			STAI–Trait			Pittsburgh			Yoshitake		
	β	95% CI	*p*	β	95% CI	*p*	β	95% CI	*p*	β	95% CI	*p*	β	95% CI	*p*
Sex															
Female	3.60	−1.85, 9.05	0.192	6.74	0.16, 13.32	0.045	1.60	−5.15, 8.35	0.638	2.26	0.15, 4.38	0.036	12.67	−1.96, 27.29	0.088
Group × Institution															
1 × 1	2.39	−6.07, 10.84	0.575	−1.12	−11.33, 9.08	0.827	2.46	−8.01, 12.93	0.641	2.98	−0.30, 6.26	0.074	−5.90	−28.58, 16.78	0.605
1 × 2	0.80	−7.47, 9.07	0.847	3.45	−6.53, 13.44	0.493	2.61	−7.63, 12.85	0.613	0.43	−2.77, 3.64	0.789	−7.74	−29.93, 14.45	0.489
2 × 1	2.69	−6.30, 11.68	0.553	−0.07	−10.93, 10.78	0.989	1.31	−9.82, 12.45	0.814	2.15	−1.34, 5.63	0.224	−20.97	−45.10, 3.16	0.087
2 × 2	0.34	−7.95, 8.63	0.935	−2.63	−12.63, 7.38	0.602	−2.54	−12.81, 7.72	0.623	−1.11	−4.33, 2.10	0.491	−25.70	−47.93, −3.46	0.024

95% CI: 95% confidence interval. Intervention groups: 1 = Metropolitan Parks, 2 = Urban Forest. Institution groups: 1 = Institution A, 2 = Institution B. The control group was used as the base of comparison in the group × institution interaction, and for the sex variable, males were established as the base group for comparison. *p*-values were obtained using a multivariate regression model. *p* < 0.05 were considered statistically significant.

**Table 4:** A multilevel mixed-effects linear regression model between CAR concentration and the interaction between the intervention group and time of intervention.

Variable	Coefficient	95% CI	*p*
Groupxtime			
Control	1.20	0.11, 2.28	0.03
Parks	1.70	072, 2.67	<0.01
Forest	1.74	0.73, 2.76	<0.01

95% CI: 95% confidence interval. *p*-values were obtained using multilevel mixed-effects linear regression. *p* < 0.05 were considered statistically significant.

## Data Availability

The data presented in this study are available upon reasonable request to the corresponding author.

## Supplementary Materials

The following supporting information can be downloaded at https://www.mdpi.com/article/10.3390/ejihpe15080159/s1; Figure S1: Report on temperature and relative humidity for each intervention week in the metropolitan park (a) and urban forest (b) groups; Table S1: Guidelines for assessing the CAR considered in this study.

## Abbreviations

The following abbreviations are used in this manuscript:

CAR	Cortisol Awakening Response
NIH	National Institutes of Health
CEMIN	Ethics and Research Methodologies Committee
PSS-14	Perceived Stress Scale
STAI	State-Trait Anxiety Inventory
PSQI	Pittsburgh Sleep Quality Index
MANOVA	Multivariate Analysis of Variance
BMI	Body Mass Index
HPA	hypothalamic–pituitary–adrenal
