# Supplementary material for: Promoting Healthy Organizations Through Urban Nature: Psychological and Physiological Effects in Healthcare Workers
Source: Eur J Investig Health Psychol Educ. 2025 Aug 14;15(8):159. doi: 10.3390/ejihpe15080159 (PMC12385907; doi:10.3390/ejihpe15080159)
Supplement: Supplementary file 1 [file ejihpe-15-00159-s001.zip › ejihpe-3527021-supplementary.pdf]

Supplementary Materials

Table S1. Guidelines for assessing the CAR considered in this study

Control of sampling accuracy<br>- Use a self-report diary system to complement objective data<br>This self-reporting system was implemented through the recording of the time at which each saliva sample was collected by the participant, complemented by photographic records specifying the collection time.
Participant instructions<br>- Inform participants about the use of objective monitoring strategies<br>- Consider employing additional strategies for maximizing adherence (provide additional take-home instructions, specifically emphasize the importance of collecting S1 immediately upon awakening, allow participants to ask questions, and reminder text messaging through research personnel the evening before sampling).<br>- Provide clear instructions about unwanted morning behavior<br><br>To ensure accurate sample collection, the research team conducted small-group sessions with participants, explaining the proper collection procedures and emphasizing their importance in preventing errors in the results. Additionally, step-by-step written instructions were provided with the collection tubes, along with a video detailing the correct procedure for sample collection. These instructions included guidelines on avoiding undesirable behaviors such as smoking, exercising, or consuming any food before sample collection. Finally, participants received a text message on their cell phones the night before to remind them of the collection process and reinforce key instructions.
Influence of covariates<br>- Assess and control for state and trait covariates.<br>- In cross-sectional research: Make sure the study design limits the possibility of systematic differences in the examination context (weekday-weekend differences)<br>- Postpone sampling in case of acute illness or circadian disruptions<br><br>In the study, state variables were considered, such as the time of awakening, the duration of sleep from the previous day, the quality of sleep was assessed, and sample collection was conducted only on weekdays. Additionally, the study collected information on trait variables such as age, sex, socioeconomic status, current smoking, alcohol consumption, BMI, and the use of oral contraceptives.<br>In addition, when a participant reported having an acute illness, sample collection was postponed until the participant reported improvement.

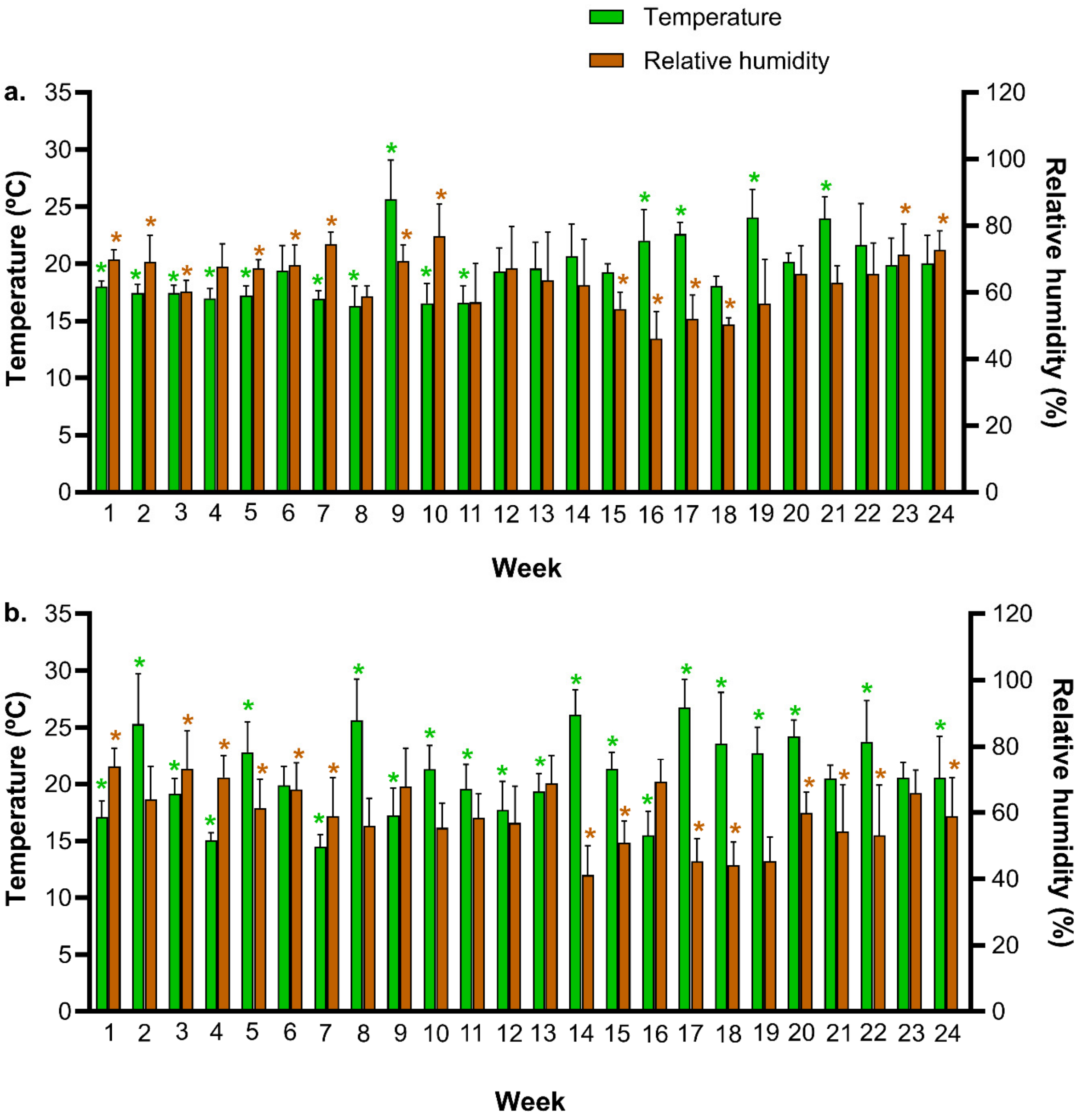

Figure S1. Report on temperature and relative humidity for each intervention week in the metropolitan park (a) and urban forest (b) groups. *Measurements that exhibited significant differences compared to at least two other time points within each intervention group. *p* values were obtained using the Kruskal-Wallis test, followed by Dunn's post hoc test. A $p<0.05$ was considered statistically significant.
